# Innovative textiles treated with TiO_2_-AgNPs with succinic acid as a cross-linking agent for medical uses

**DOI:** 10.1038/s41598-024-56653-7

**Published:** 2024-04-05

**Authors:** Mohamed Abdel-Shakur Ali, Emam Abdel-Mobdy Abdel-Rahim, Azza Abdel-Aziz Mahmoud, Sahar Emam Mohamed

**Affiliations:** 1 Biochemistry Department, Faculty of Agriculture, Cairo University, Giza, Egypt; 2 Cotton Technology Research Division, Cotton Research Institute, Agriculture Research Center, Giza, Egypt

**Keywords:** Silver nanoparticles, Titanium dioxide nanoparticles, Core-shell, UV protection, Self-cleaning, Antimicrobial, Textiles and cotton fabrics, Biochemistry, Microbiology, Plant sciences, Nanoscience and technology

## Abstract

Silver and titanium-silver nanoparticles have unique properties that make the textile industry progress through the high quality of textiles. Preparation of AgNPs and TiO_2_-Ag core–shell nanoparticles in different concentrations (0.01% and 0.1% OWF) and applying it to cotton fabrics (Giza 88 and Giza 94) by using succinic acid 5%/SHP as a cross-linking agent. Ultra-violet visible spectroscopy (UV–Vis), X-ray diffraction (XRD), dynamic light scattering (DLS), zeta potential, transmission electron microscopy (TEM), scanning electron microscopy/energy-dispersive X-ray (SEM–EDX) are tools for AgNPs and TiO_2_-AgNPs characterization and the treated cotton. The resulting AgNPs and TiO_2_-AgNPs were added to cotton fabrics at different concentrations. The antimicrobial activities, UV protection, self-cleaning, and the treated fabrics' mechanical characteristics were investigated. Silver nanoparticles and titanium dioxide-silver nanoparticles core–shell were prepared to be used in the treatment of cotton fabrics to improve their UV protection properties, self-cleaning, elongation and strength, as well as the antimicrobial activities to use the produced textiles for medical and laboratory uses and to increase protection for medical workers taking into account the spread of infection. The results demonstrated that a suitable distribution of prepared AgNPs supported the spherical form. Additionally, AgNPs and TiO_2_-AgNPs have both achieved stability, with values of (− 20.8 mV and − 30 mV, respectively). The synthesized nanoparticles spread and penetrated textiles' surfaces with efficiency. The findings demonstrated the superior UV protection value (UPF 50+) and self-cleaning capabilities of AgNPs and TiO_2_-AgNPs. In the treatment with 0.01% AgNPs and TiO_2_-AgNPs, the tensile strength dropped, but the mechanical characteristics were enhanced by raising the concentration to 0.1%. The results of this investigation demonstrated that the cotton fabric treated with TiO_2_-AgNPs exhibited superior general characteristics when compared to the sample treated only with AgNPs.

## Introduction

Commercialization applications of nanoparticles, particularly self-cleaning and antibacterial technical textiles, offer enormous potential for the technical textile sector. By incorporating metal and metal oxide nanoparticles into textile finishing operations, such nano-based high-performance textiles may be created right away^1,2^. Metallic NPs have tremendous potential that may be effectively used to create useful fabrics. Metallic-based particles can provide intriguing finishes without detracting from the fabrics' look or negatively affecting their properties. According to numerous studies^3–5^ textiles can be treated with silver, titanium dioxide, zinc oxide and silicon dioxide nanoparticles as surface-modifying agents to add various properties like ultraviolet protection, self-cleaning, and antimicrobial activities. The treated cotton fabric showed durable super hydrophobicity, dye degradation, and antibacterial activity with UPF more than 90 up to 20 industrial laundering cycles. The highest water contact angle obtained was 150.5° which was reduced to 131° after 20 washing cycles. The treated fabric exhibited good antibacterial activity against bacteria (*S. aureus* and *E. coli*) even after 20 industrial laundering cycles showing good durability of NPs without having any significant effect on physical properties of fabric after treatment (3–5). Recently, it has been reported that titanium dioxide nanoparticles may be adhered to fabric surfaces using a variety of techniques such as drop casting, dip coating, optical deposition, layer-by-layer deposition, electrospin and electrosprayingto create a self-cleaning surface. Among these techniques, drop casting and dip coating are the simplest ones, whereas optical deposition allows monitoring the process of coating, layer-by-layer depositionis time-consuming technique, and electrospin/electrospraying technique required special equipment^6^.

It has been demonstrated that titanium dioxide nanoparticles have photocatalytic activity. The ability of a valence band electron to be driven to the conduction band and produce pairs of electrons (e) and holes (h) occurs when TiO_2_NPs are exposed to UV radiation (l 388 nm). These reactive species play a crucial role in starting a redox interaction^7^.

Titanium dioxide has been identified as a suitable photocatalytic agent in the presence of UV radiation due to its low cost, non-toxicity, chemical and physical stability, and favorable optical properties. The large band gap (3.2 eV) and the fact that it can only be stimulated by UV radiation (388 nm) to discharge electrons to the conductive band while leaving holes in the valence band are some drawbacks connected with its use. Consequently, this limits the titanium dioxide photocatalytic reaction process's ability to use sunlight or other visible light as an external excitation source. "Additionally, the high rate of hole/electron recombination in TiO_2_NPs leads to less effective photocatalysis^8,9^".

Silver nanoparticles are one of the most promising nanomaterials for commercial uses^10,11^. Numerous applications, such as the sterilization of medical equipment, household appliances, and the purification of water, have made use of silver nanoparticles as antimicrobial agents. "They have a wide range of applications, including electrical goods, antibacterial agents in hospitals, food storage, textile coatings, wound healing applications, and several green purposes^12–17^". It has been examined and shown that Ag coated on titanium dioxide film using the chemical vapor deposition approach exhibits superior photocatalytic activity than other catalysts. The noble metal deposition method was widely employed to increase the photocatalytic activity of TiO_2_^18–20^. Ag cannot aggregate due to the core-shell structure. TiO_2_-AgNPs showed much higher photocatalytic activity in the methylene blue degradation process when compared to pure TiO_2_. According to XPS research, the TiO_2_-AgNPs film has more surface hydroxyl groups than the TiO_2_ film^21^. The surface OH groups were transformed into OH^•^ radicals during UV irradiation, which was essential for photocatalytic activity^21^.

Core-shell composites structures, as a kind of new nanostructures, have received intense attention due to their improved physical and chemical properties over their single components, and thus many efforts have been made to synthesize such special core-shell nanostructures. The core-shell composites have many advantages as antibacterial agent, low toxicity, chemical stability. The current study aimed to synthesize and characterize silver nanoparticles and TiO_2_-AgNPs core-shell and to treat the cotton fabrics with prepared nanoparticles using succinic acid as cross-linking agent in order to increase the efficiency of the textiles to UV protection and increase the self-cleaning ability to be used in producing textiles for medical uses.

## Material and methods

### Cotton fabrics

The unbleached long stable Egyptian cotton fabrics were obtained from Miser-El-Mehala Company for Spinning and Textile, Egypt, which were made from Giza 88 and Giza 94. The fabrics are plan weaved with a warp of 36 yarns per cm^2^ and a weft of 30 yarns per cm^2^. All experimental materials were under controlled atmospheric conditions of 20 ± 2 °C temperature and 65 ± 2% relative humidity.

### Chemicals

Titanium (IV) isopropoxide, + 97% (C_12_H_28_O_4_Ti) was obtained and purchased from Thermo Fisher (Kandel), Germany. Silver nitrate was purchased from Sigma Aldrich. Silver nitrate powder 99.99% was purchased from Sigma Aldrich., Soluble starch, succinic acid 99%, NaOH and H_2_O_2_ were purchased from PIOCHEM. Sodium hypophosphate (SHP) was obtained from Central Drug House (P) LTD New Delhi. Reactive (drimen) dye blue 2 (C_29_H_20_ClN_7_O_11_S_3_ was purchased from ICN biomedical.INC, Germany.

### Pretreatment processes

#### Scouring and bleaching with NaOH and H_2_O_2_

Non-cellulosic material on fabrics was removed by saturate fabric at 100 °C for 90 min with one liter of aqueous solution containing 40 g/L NaOH (1M) and 1% mercerol (wetting agent) with constant stirring. The liquor ratio will keep being 1:30. After scouring, the treated samples were washed under running water and dried. The scoured fabrics were bleached to remove colored materials following the recipe silicate (Na_2_SiO_3_·9H_2_O) 3.5%, Na_2_CO_3_ 1% and H_2_O_2_ 4% for 1 h at 90 °C. All the desiring, scouring and bleaching experimental were carried out in triplicate at liquor ratio of 20:1^22^.

#### Dyeing process

The modified fabrics were dyed with 1% owf (on weight of fabric) reactive (drimen) dye with 6% (owf) at 1:50 LR (material to liquor ratio), Firstly the samples were immersed in the water and dyeing bath was warmed at 50 °C, then added salt to the dyeing bath into three times followed by adding the dye solution, then the temperature was raised to boiling through 15 min, and the dyeing was continued at this temperature for 45 min, finally the dyeing was stopped and the dyeing bath was cooled. Dyed samples were thoroughly rinsed with running water, then soaped with a solution containing 5 g/L nonionic detergent (Hostapal CV-ET) at 70 °C for 15 min, Finally, the samples were rinsed with water after washing, and left to dry in air^23^.

### Treatment processes

#### Silver nanoparticles preparation (AgNPs)

In the chemical reduction technique, alkali-dissolved starch was used as a stabilizing agent for the produced silver nanoparticles and as a reducing agent for silver ions. AgNPs were created using a high-speed homogenizer and a thermostatic water bath. Using a high-speed homogenizer, 5 g of native maize starch were completely dissolved in 80 mL of distilled water with 1.5 g of sodium hydroxide to create an alkali-dissolved starch solution. The mixture was fully dissolved before being heated to 70 °C. AgNO_3_ (0.01 and 0.1 g) dissolved in 20 mL of distilled water were mixed with an aqueous solution (pH 12) of alkali-dissolved starch, drop by drop. For 60 min, the reaction mixture was continuously stirred. AgNO_3_ solution was added, and shortly after, the reaction medium turned clear yellow, then brown, and finally dark brown, confirming the creation of AgNPs^13^. The colloidal solution was allowed to cool gradually to 25 °C during a 30-min period following the completion of the reaction^22^.

#### Titanium dioxide nanoparticles preparation (TiO_2_NPs)

Using a magnetic stirrer, titanium tetra isopropoxide (6 mL) was combined with 1% acetic acid (2 mL). Following the addition of 56 mL of ethanol dropwise with constant stirring, the solution's pH was then corrected by adding concentrated HCl (2 mL) after 5 min. The mixture was thoroughly magnetically swirled for 45 min. after which a viscous solution was produced, indicating that TiO_2_-NPs sol-gel had formed. This process was followed by 24 h of drying at 110 °C and 2 h of calcination at 450 °C in a muffle furnace^24^.

#### Preparation of TiO_2_-Ag core–shell nanoparticle

With a few minor modifications, the core–shell type TiO_2_-Ag was made by the method of Neil et al.^25^. In a nutshell, 30 mL of distilled water was used to sonicate 1 g of TiO_2_-NPs with a 50 nm average size for 10 min. The silver nitrate solution used had the proper concentration. To create a partly stabilized dispersion, the required quantity of AgNO_3_ was dissolved in deionized water (0.01–0.1 g AgNO_3_ in 500 mL DW). After that, 10 g of gelatin was added to 30 mL of the TiO_2_ solution and then sonicated for 10 min. The dispersions were dried at 110 °C for 24 h, and then the powder samples were subjected to a 2-h calcination process at a specified temperature of 450 °C.

#### Cotton fabric treatment with AgNPs and TiO_2_-AgNPs

5% wt/wt succinic acid and 4% wt/wt sodium hypophosphite were mixed in the finishing bath with 100 mL volume. to crosslink Ag and TiO_2_-AgNPs on cotton fabric samples. To create a dispersed solution, the prepared solution of Ag and TiO_2_-AgNPs (0.01% and 0.1% OWF) was sonicated for 20 min. At room temperature, the cotton materials were submerged in the solution. The treated materials were then cured for 1.5 min. at 150 °C after being dried for 3 min at 120 °C. These remedies underwent twofold drying and curing^26^.

### Characterization of nanoparticles

#### UV–Vis spectroscopy

The optical properties of AgNPs and TiO_2_-AgNPs were measured using a PG Instruments Ltd. T80 UV–Vis spectrophotometer in the Cotton Research Institute, Agriculture Research Centre, Egypt. At wavelengths between 300 and 700 nm, UV–Vis spectra were captured.

#### Transmission electron microscopy (TEM)

AgNPs and TiO_2_-AgNPs were screened morphologically using transmission electron microscopy (JEM-1400, JEOL model). The imaging was captured by the electronic microscope lab at Cairo University Research Park (CURP). TiO_2_-AgNPs and freshly prepared AgNPs were placed on a glow-discharged carbon grid. Then, the shape and the diameter of the nanoparticle specimens were determined by the TEM operating system.

#### Particle size and zeta potential

Using a Zetasizer^®^ 3000 particulate size description analyzer (Malvern Instruments) in the central lab of National Institute of Standards (NIS), a complementary scientific research institute with analytical services, and by using photon interconnection spectroscopy and Laser Doppler intensity measurement, the zeta potential and particle size of the nanoparticles were ascertained, respectively. The mean hydrodynamic diameter was determined by progressive analysis after size correction was carried out three times at a 25 °C scattering angle and each correction was time-restricted for 3 min. The automated water dip cell mode was used to check the zeta potential adjustment.

### Characterization of TiO_2_NPs coated with cotton fabrics 

#### Scanning electron microscopy (SEM)

The imaging of nanoparticles was captured by SEM Quanta 250 FEG (Field Emission Gun) connected to an EDX unit (energy dispersive X-ray analyses) at 30 kV accelerating voltage and 14× to 1 million magnification.

#### X-ray diffraction (XRD)

The nanoparticle on the textiles was examined using X-ray diffraction (XRD). CuK radiation and = 1.5406 A were used to capture XRD patterns using a Bruker, D8 advance rotaflex diffraction meter. For cellulose Crystallinity, the following height ratio was used to compute the XRD crystallinity index (CIXRD), based on the peak height method for native cellulose developed by Segal et al.^27^:

$${{\text{CI}}_{{{\text{XRD}}}} \left( \% \right) = \frac{{{\text{I}}_{002} - I_{am}}}{I_{002}} \times 100}$$

where I_002_ was the intensity of the crystalline and I_am_ the height of the minimum.

### Application of TiO_2_NPs on cotton fabrics

#### UV protection

Using a Cary 50 solar screen transmission spectrophotometer, the transmissions of UV radiation were measured by S.A.A.S^28^, Sun protective apparel-evaluation and classification. UPF values were determined automatically by Sharma and Singh^29^.

The amount of UV light that passes through a substance affects its UPF. UPF measures how much longer a person can be exposed to the sun while wearing a certain garment before their skin starts to get red^30^.

#### Anti-bacterial screening test

The agar plate technique^31^ was used as a qualitative test with two microorganisms, Gram-negative Escherichia coli (ATCC 8739) and Gram-positive *Staphylococcus aureus* (ATCC 6538), to determine if the substances had an antibacterial effect.

#### Agar plate method

In sterile Petri plates, the bacteriostatic activity was administered. *E. coli and S. aureus* 24-h broth cultures, the test organisms, were employed as inoculums. The test organisms were swabbed over the agar plates' surface using a sterile cotton swab. The core of the mat culture was gently pressed with the test textiles (fabrics coated with nanoparticles) and the control fabrics. For 18–24 h, the plates were incubated at 37 °C.

#### Self-cleaning property

Testing and self-cleaning finishes fabric samples that had not been treated as well as those that were loaded with Ag and TiO_2_-AgNPs were stained for 15 min in a solution of basic blue dye (1% OWF), dried, and then conditioned. For 24 h, the samples were exposed to light from the sun. Fabric samples loaded with Ag and TiO_2_-AgNPs as well as untreated samples had their color strength represented as a K/S value evaluated. The deterioration of the dye stains directly causes the drop in K/S values^24,32,33^.

#### Strength and elongation

The force per unit linear density of the unstrained specimen is used to express the tensile stress. The National Institute for Standard (NIS-Egypt)'s Textile Metrology Department used a Shimadzu Testing Machino-Japan to test the tensile strength (Kg f) and elongation (%).

## Results and discussion

### Characterization of silver nanoparticles (AgNPs) and titanium oxide-silver nanoparticles (TiO_2_-AgNPs)

#### Transmission electron microscope (TEM) of AgNPs and TiO_2_-AgNPs

**Figure 1:**
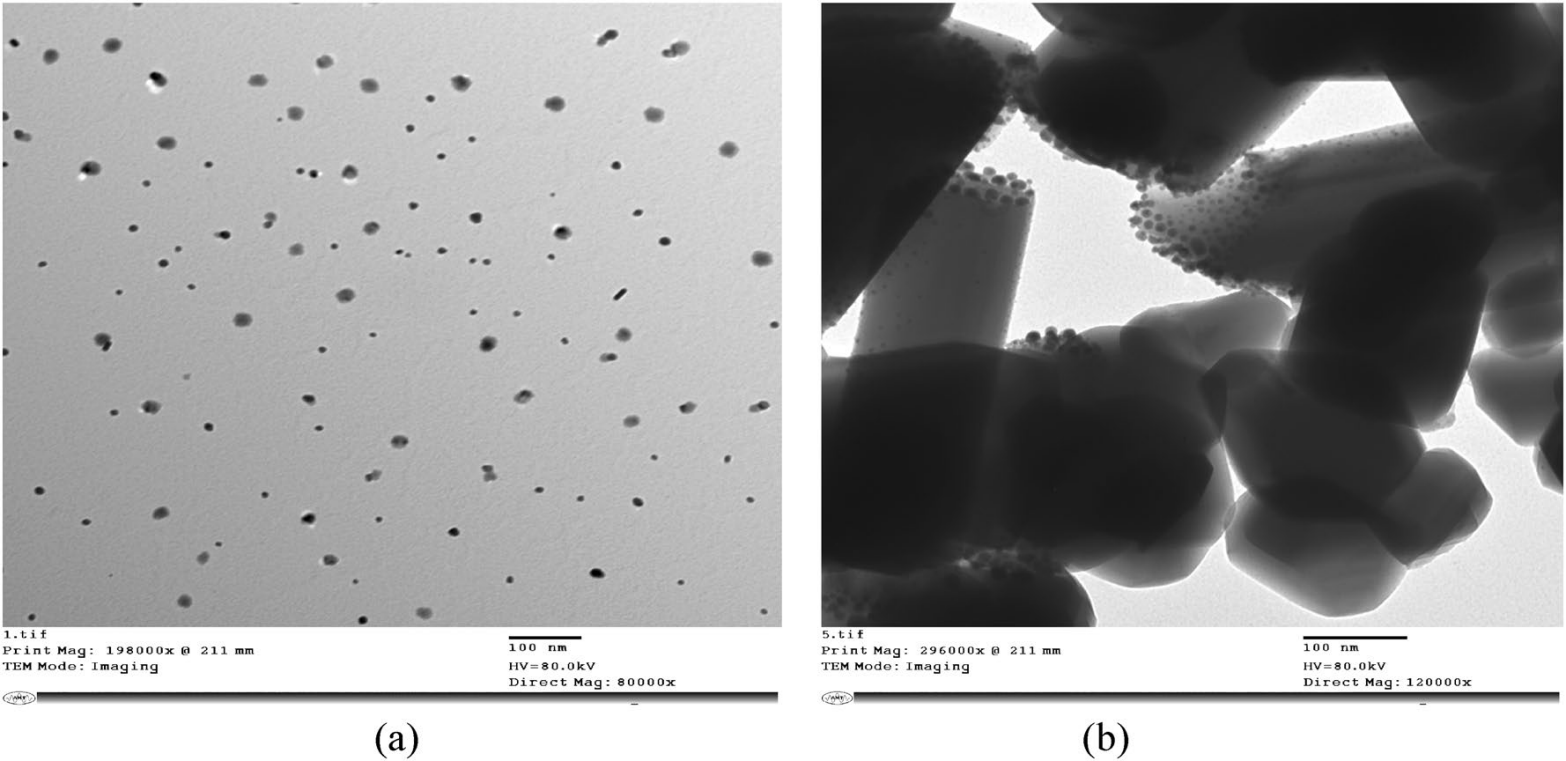
Transmission electron microscope micrograph of (**a**) AgNPs and (**b**) TiO_2_-AgNPs.

Figure 1a shows the TEM of the prepared AgNPs by reduction method. The size and shape of the silver nanoparticles are homogeneously monodispersed. The NPs were observed to be irregular and spherical and the size range from 10.4 to 22.2 nm. These results are in agreement with Hebeish et al.^34^, who observed that the TEM of AgNPs has a spherical morphology and the size of AgNPs lies between 7.53 and 21.060 nm.

Figure 1b shows the TEM of the TiO_2_-Ag core–shell. It is evident that the TiO_2_ serves as the shell and the silver nanoparticles serve as the core; both have uniform hexagonal shapes with size range from 39.7 to 74.6 nm. The average core size is 11.54 nm, and the average shell thickness is 94.5 nm. These findings concur with those of Yang et al.^35^ and Min et al.^36^, they obtained that TEM images of Ag-TiO_2_ core-shell nanoparticles have Ag cores with diameters of 50–100 nm generated by a polyol-thermal approach.

#### UV–Vis spectrum of AgNPs and TiO_2_-AgNPs

**Figure 2:**
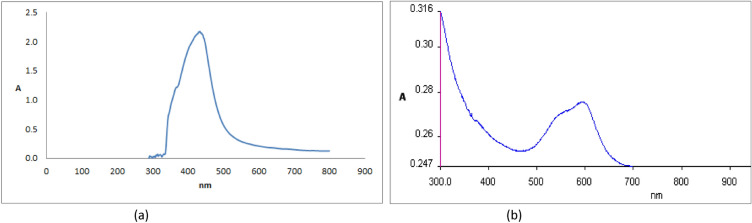
UV–Vis absorption spectra of (**a**) AgNPs and (**b**) TiO_2_-Ag core-shell nanoparticle absorbance.

The optical characteristics of nanoparticles were evaluated using a UV–Vis spectrometer. The UV–Vis spectra for the core–shell architectures of AgNPs and TiO_2_-AgNPs are shown in Fig. 2. When coated with TiO_2_ nanoparticles, the wavelength absorbance peak of the Ag nanoparticles, which is centered at 440 nm (Fig. 2a), is attenuated. It also moved in the absorption wavelength area (316 nm) in Fig. 2b, which is comparable to that of Ag-TiO_2_NPs. These results agree with Yang et al.^35^ and Min et al.^36^ which found that the wavelength peak of TiO_2_-AgNPs at 320 nm.

#### Energy dispersive X-ray spectra (EDX) of AgNPs and TiO_2_-AgNPs

The composition of the Ag and TiO_2_-Ag nanoparticles was determined by energy dispersive X-ray (EDX). The produced AgNPs were subjected to chemical analysis using EDX, which verified the presence of both Ag and the alkali-dissolved starch coating the AgNPs. Metallic silver nano-crystals typically exhibit an optical observation peak at about 2.984 keV because of surface plasmon resonance.

**Figure 3:**
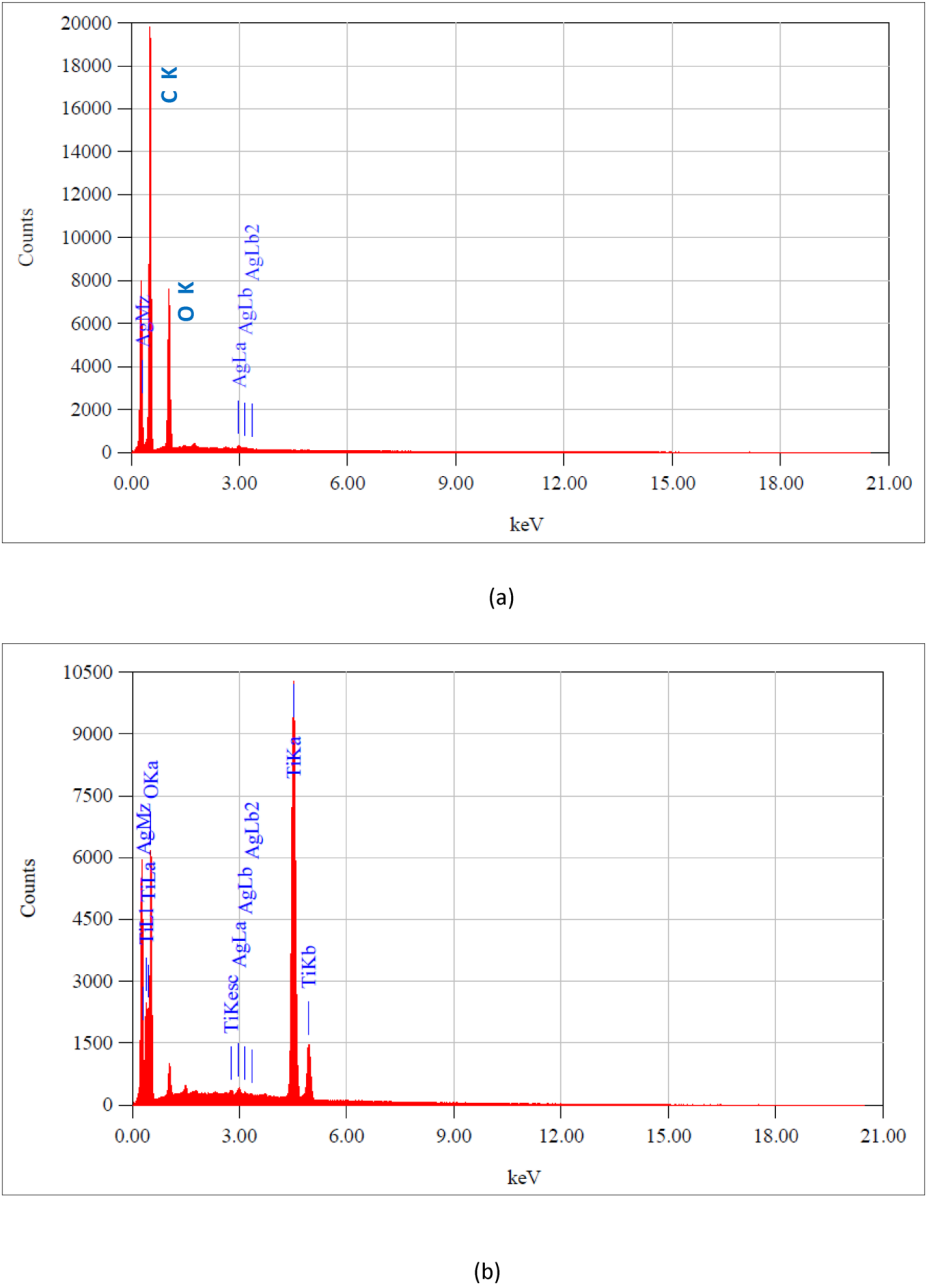
EDX spectra of (**a**) AgNPs and (**b**) TiO_2_-Ag core–shell nanoparticle.

The Ag nanoparticles are also demonstrated by the EDX spectra to be in the metallic state, devoid of Ag_2_O production and other contaminants. Figure 3a provided evidence of the high concentration of powdered AgNPs. AgNPs have a molecular ratio of 100%. These findings concur with those of Hebeish et al.^34^, who found that surface plasmon resonance causes metallic silver nanocrystals to typically exhibit an optical observation peak at 3 keV. The spectrum of TiO_2_-AgNPs, as depicted in (Fig. 3b), clearly shows prominent peaks of the components Ag, Ti, and O, demonstrating that the TiO_2_ layer successfully coated the Ag nanoparticles. Ag, Ti, and O have molecular ratios of 0.95%, 67.01%, and 32.04%, respectively according to Min et al.^36^, which found that the Ag, Ti, and O all exhibit prominent peaks in the spectrum, indicating that the TiO_2_ layer coating of the Ag nanoparticles was effective.

#### Particle size (PS) and zeta potential (ZP)

**Figure 4:**
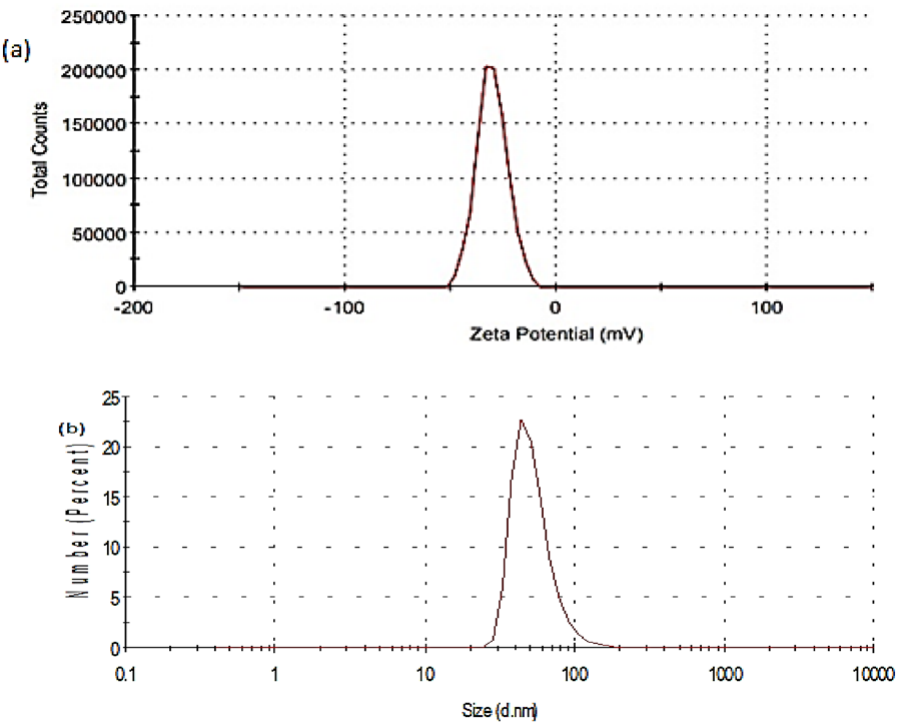
(**a**) Zeta potential and (**b**) size distribution by number of AgNPs.

**Figure 5:**
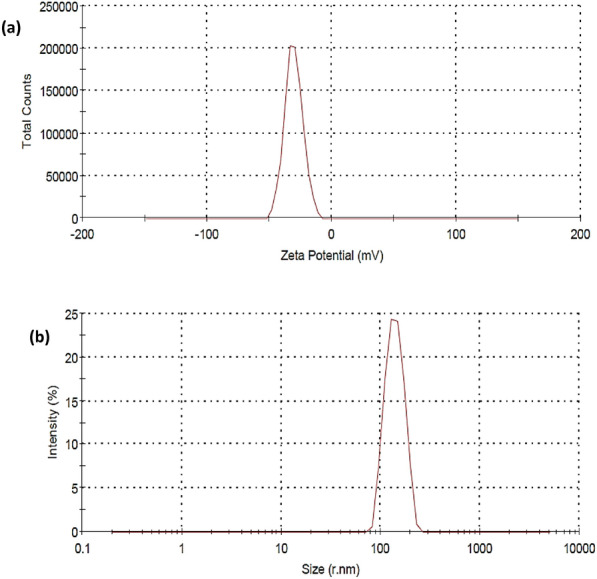
(**a**) Zeta potential and (**b**) size distribution by number of TiO_2_-AgNPs core–shell.

The size and charge of nanomaterials can be determined by zetasizer. Figures 4 and 5 show the particle size distribution and zeta potential of AgNPs and TiO_2_-AgNPs core-shell. It’s clear in Fig. 4a,b the size distribution of the colloidal AgNPs with a concentration of 0.01% was found at 120 nm, and the ZP value was determined to be − 20.8 mV. The presence of hydroxyl groups from starch molecules structure cap the produced AgNPs, which results in their negative ZP according to Hebeish et al.^34^. The average size and zeta potential of core-shell nanoparticle TiO_2_-AgNPs are presented in Fig. 5a,b which show The size of the colloidal TiO_2_-AgNPs is 140.2 nm and the ZP of these colloidal is (− 30.7 mV). These results are in agreement with Cosgrove^37^, which found that the solutions with zeta potential above + 20 mV or below − 20 mV are stable.

### Cotton fabrics characterization

The main aim of this work is to apply AgNPs and TiO_2_-AgNPs to cotton fabric (G88 and G94) as an antibacterial finishing agent, UV protection, and self-cleaning agent. The treated cotton fabric was then assessed.

#### Scanning electron microscope (SEM)

**Figure 6:**
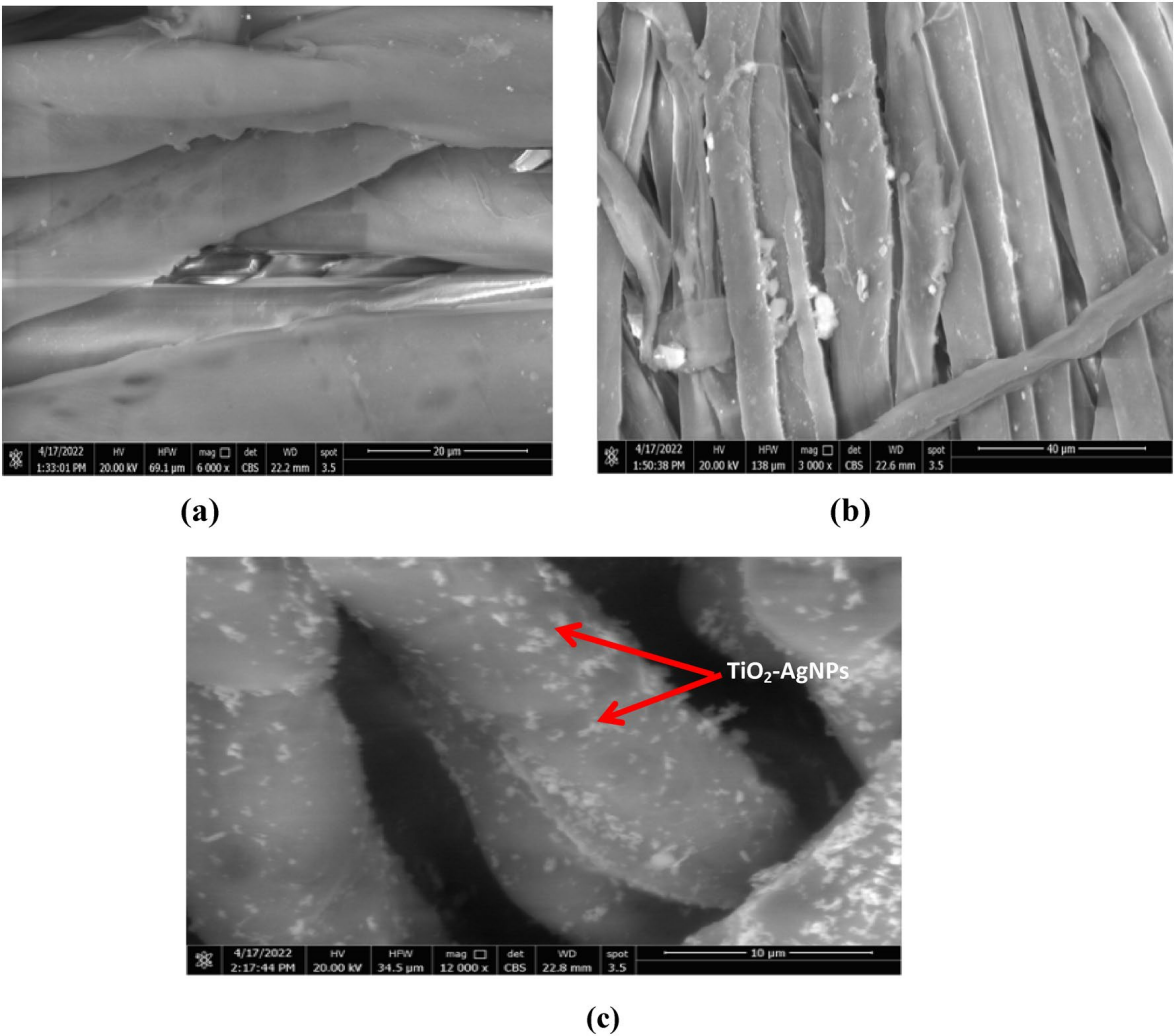
SEM of cotton fabric samples coated with TiO_2_-Ag core–shell (**a**) untreated cotton (**b**) treated with AgNPs (**c**) treated with TiO_2_-AgNPs core–shell.

Investigations were conducted into the morphological structure of cotton textiles treated with AgNPs and TiO_2_-AgNPs, respectively, and compared to untreated cotton fabric. Figure 6 depicts the surface structure of cotton fabric both before and after treatment. Figure 6a shows that the cotton fabric is untreated and has smooth sheaths around the tangled textiles with no discernible particles accumulated on the surface. As a result of the textiles being treated with prepared nanomaterials (AgNPs and TiO_2_-AgNPs), (Fig. 6b,c) exhibits a rough surface. To better understand the situation, a high-magnification SEM image of the treated textiles is obtained to show the morphology of the deposited particles from the two distinct materials (Fig. 6b,c). The particles that developed on the surface of textiles seem to be spherical at this high SEM magnification, and the nanoparticles are evenly spread over the fiber surface. these results in agreement with Yang et al.^38^ and El‐Naggar et al.^39^, they found that the morphological structure analyses confirm that the cotton fabric's inherent properties allow nanoparticles to be spontaneously captured and settled onto the fabric.

#### X-ray diffraction studies

**Figure 7:**
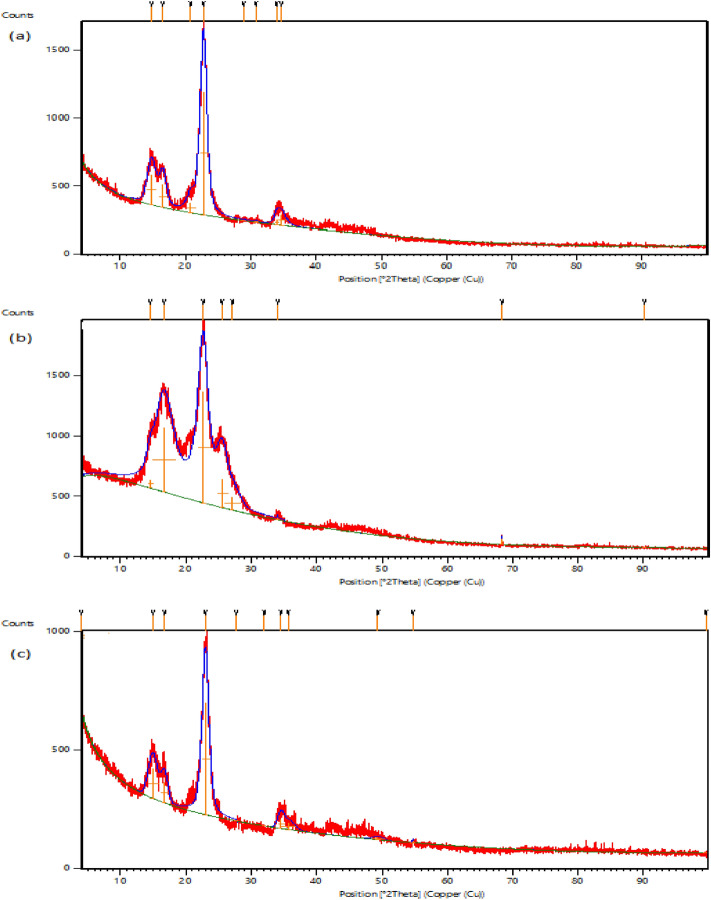
XRD studies of cotton fabric samples G88 (**a**) untreated cotton (**b**) treated with AgNPs (**c**) treated with Ag-TiO_2_NPs.

**Figure 8:**
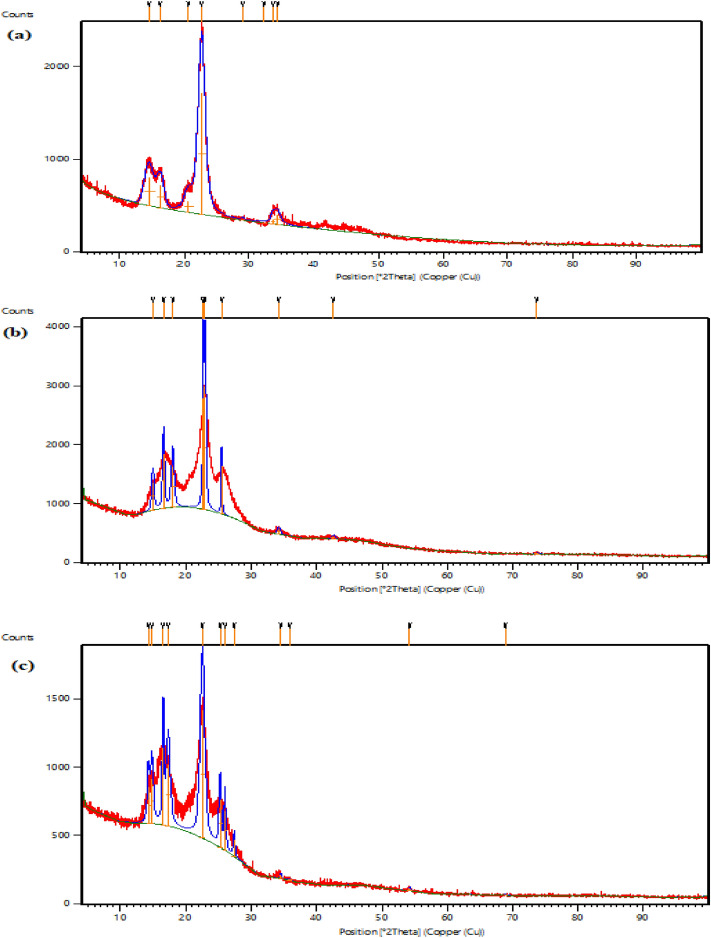
XRD studies of cotton fabric samples G94 (**a**) untreated cotton (**b**) treated with AgNPs (**c**) treated with Ag-TiO_2_NPs.

The crystalline structure of the cotton coated by AgNPs and TiO_2_-AgNPs was examined using the XRD pattern. Figures 7a-c and 8a-c show the XRD of cotton fabric G88 and G94, respectively. All samples' XRD spectra show two distinct peaks at 2 angles of 16.450 and 22.640 that originate from cotton as the primary substrate (control sample in Figs. 7a, 8a). The presence of AgNPs on the treated textiles (Figs. 7b, 8b) can be confirmed by characterization peaks at 2θ angles 34.21°, 42.580°, and 73.69°. Characterization peaks at 2θ angles in the spectra of the cotton samples treated with TiO_2_-Ag nanocomposites (Figs. 7c, 8c) provide evidence for their existence, 25.25°, 25.98°, 27.40°, 34.51°, 35.89°, 54.25° and 68.84° and in agreement with Yang et al.^36^, who found that the XRD pattern of Ag/TiO_2_ and exhibits characteristic peaks at 2θ 25.37°, 48.12°, 53.97° and 55.10°, corresponding to (101), (200), and (105) planes, respectively, which belong to typical anatase-TiO_2_ materials. The crystallinity index of the fabrics treated with AgNPs was increased from 76.40 to 92.33%. On the other hand, the treated fabrics with core-shell Ag-TiO_2_ was 81.35% in the case of G88, and the crystallinity index of G94 was increased from 75.42% to 77.33 in concentration of 0.1% AgNPs but decreased in the case of core–shell TiO_2_-Ag to 71.93%.

#### UV protection property

It is generally known that prolonged exposure to UV radiation may cause serious damage to human skin. These UV rays have been connected to skin cancer development. The UV–Visible spectrophotometer was used to measure the treated cotton fabric's capacity to block UV radiations or UPF.

**Figure 9:**
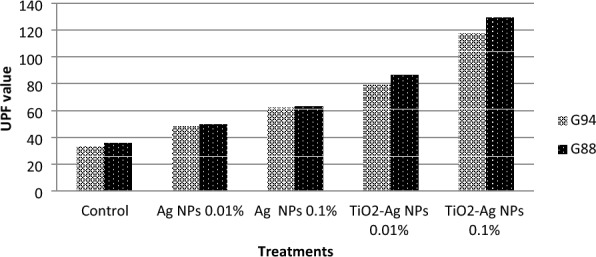
Effect of AgNPs and TiO_2_-AgNPs core–shell with succinic acid on UV protection of cotton fabrics.

The results in Table 1 and Fig. 9 show the application of AgNPs and TiO_2_-AgNPs core-shell nanoparticles with succinic acid / SHP cross linking agents in different concentrations (0.01% and 0.1% OWF). Table 2 displays the UVA rays transmitted for cotton fabrics in the UVA (320–400 nm) and UVB (280–320 nm) regions (abbreviated as TUVA and TUVB). UV transmission on treated cellulose fabrics (G88 and G94) was significantly lower than on untreated cellulose fabrics, which led to an increase in the UPF (Ultraviolet Protection Factor) value that was automatically calculated, this number indicates how long a person can stay in the sun while wearing protective clothes before their skin starts to get red. The results show that G88 indicates a higher UPF value followed by G94 and core-shell nanoparticles give high results compared with silver only owing to the dual effect of AgNPs and TiO_2_-Ag nanoparticles. TiO_2_-Ag nanoparticles, which have a high photocatalytic activity, a low density, and an excellent resistance to UV radiation, can be used as an example of this phenomenon. All treated cotton fabrics give excellent UV protection. These results in agreement with Ali et al.^40^ and Jafari-Kiyan et al.^41^ they found that after functional finishing of cotton by titanium dioxide nanoparticles, the transmittance values of the sample decreased significantly because of the UV absorption ability of TiO_2_-NPs. Moreover, the UV-blocking activity of the nanocomposites-treated fabrics was improved by them presence of silver nanoparticles on the surface of cotton fabrics. Due to the UV reflection ability of the AgNPs, coating cotton fabric with TiO_2_-Ag nanocomposites led to a more significant decrease in UV transmittance values^40^. The UPF value of the blank cotton sample was 11.12. An UPF value of 15 indicated no protection against transmittance of UV radiation through fabric onto skin. The UPF values of the treated cotton samples were measured to be 244.11, 263.23, 265.18 and 267.01 for TiO_2_ nanoparticles, yellow-nanocomposite, blue-nanocomposite, and brown-nanocomposite, respectively. Therefore, results confirmed the excellent UV-blocking activity^41^.

**Table 1:** Effect of AgNPs and TiO_2_-AgNPs core–shell with succinic acid on UV protection of cotton fabrics.

Varieties	Giza 94			Giza 88		
Treatments	UPF value	TUVA	TUVB	UPF value	TUVA	TUVB
Control	33.33 ± 0.88	7.29	3.03	35.86 ± 2.20	5.6	3.15
AgNPs 0.01%	48.25 ± 1.18	2.98	2.09	50.04 ± 1.16	1.65	2.06
AgNPs 0.1%	62.30 ± 1.17	2.59	1.8	63.44 ± 1.16	3.05	2.75
TiO_2_-AgNPs 0.01%	79.00 ± 1.16	2.56	0.4	86.06 ± 1.16	3.09	0.99
TiO_2_-AgNPs 0.1%	117.30 ± 1.20	0.92	0.51	129.23 ± 1.18	2.74	0.58

Transmitted UVA (320–400 nm) and UVB (280–320 nm) regions which were expressed as TUVA and TUVB. *UPF* UV protection factor T U.V transmitted UV radiation %. Transmitted UVA (320–400 nm) and UVB (280–320 nm) regions which were expressed as TUVA and TUVB. *UPF* UV protection factor TUV transmitted UV radiation %

**Table 2:** UV protection and classification according to^28^.

UV protection	UPF classification	transmitted UV radiation (%)
Excellent	40, 45, 50, 50+	≤ 2.5
Very good	25, 30, 35	4.1–2.6
Good	15, 20	6.7–4.2
Non-ratable	0, 5, 10	> 6.7

Transmitted UVA (320–400 nm) and UVB (280–320 nm) regions which were expressed as TUVA and TUVB. UPF: UV protection factor TUV Transmitted UV radiation %. Transmitted UVA (320–400 nm) and UVB (280–320 nm) regions which were expressed as TUVA and TUVB. UPF: UV protection factor TUV transmitted UV radiation %.

#### Self-cleaning of fabrics

**Figure 10:**
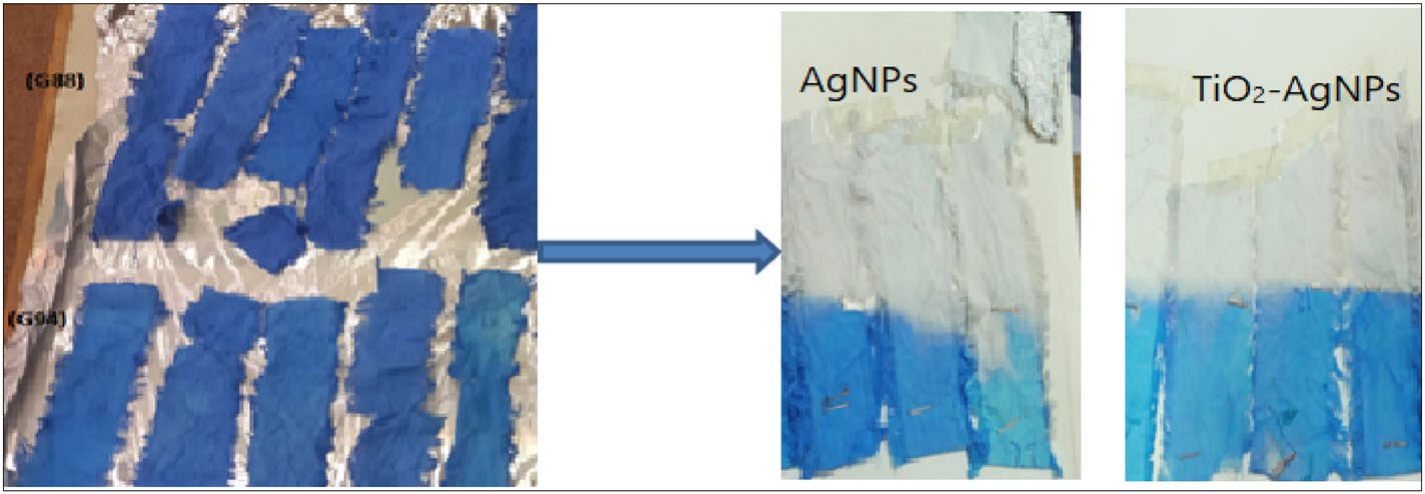
Images of degradation of methylene blue under UV radiation at 24 h by AgNPs and TiO_2_-AgNPs core-shell on cotton fabrics.

The data indicates that cotton fabrics degraded 99% of methylene blue in 24 h by photocatalysis under UV and solar irradiation, conforming to self-cleaning properties. Figure 10 shows the self-cleaning and degradation of the dye on the blank and treated cotton fabric (G88 and G94) with different concentrations of AgNPs and TiO_2_-AgNPs core–shell with different concentrations (0.01% and 0.1% OWF). A concentration of 0.01% nanoparticles resulted in the degradation of 99.9% of methylene blue. Titanium dioxide nanoparticles were used to initiate the photocatalytic breakdown of dye stains using the energy of UV radiation. By exposing the stained samples to standard laboratory settings, titanium dioxide's photocatalytic abilities changed the stains' molecular structure, making them colorless. The elimination of MB dye stains from all treated samples was found to be highly effective. The characteristics of the finished fabrics were not considerably decreased even after 50 washing cycles^42^. These findings support those of Norouzi and Maleknia^43^, Sami and Barakat^44^ and Abou-Okeil et al.^45^ who found that 2% of TiO_2_ nanoparticles are almost sufficient to give enough self- cleaning. Alvarez-Amparán et al.^46^ found that the TiO_2_-NPs functionalized cotton fabrics degraded 99% of methylene blue in 60 min by photo catalysis under UV and solar irradiation conforming self-cleaning properties.

#### Anti-bacterial properties

Tables 3 and 4 show the antibacterial properties of treated and untreated textiles with AgNPs and TiO_2_-Ag core–shell nanoparticles against common harmful bacteria like *E. coli. and S. aureus.* It has been demonstrated that the development of germs is unaffected by untreated cotton cloth. The development of the investigated microorganisms is significantly inhibited in the meanwhile by the AgNPs-treated cotton samples. Contrarily, the antimicrobial effects of textiles treated with TiO_2_-AgNPs had a greater impact against pathogenic bacteria, supporting the significance of TiO_2_NPs when bound to AgNPs. *S. aureus* has a larger inhibition zone than *E. coli,* which is lower.

**Table 3:** Effect of AgNPs and TiO_2_-AgNPs core–shell with different concentration on zone inhibition of *E.coli.*

Varieties	G88	G94
Zone inhibition (mm)		
Control	0 ± 0.00	0 ± 0.00
AgNPs 0.01%	8 ± 1.53	7 ± 1.53
AgNPs 0.1%	12 ± 2.08	10 ± 1.53
TiO_2_-AgNPs 0.01%	14 ± 1.53	11 ± 1.73
TiO_2_-AgNPs 0.1%	17 ± 1.53	13 ± 1.53

**Table 4:** Effect of AgNPs and TiO_2_-AgNPs core–shell with different concentrations on zone inhibition of *Staphylococcus aureus.*

Varieties	G88	G94
Zone inhibition (mm)		
Control	0 ± 0.00	0 ± .00
AgNPs 0.01%	11 ± 1.53	10 ± 1.53
AgNPs 0.1%	15 ± 2.73	12 ± 2.52
TiO_2_-AgNPs 0.01%	16 ± 4.16	13 ± 1.16
TiO_2_-AgNPs 0.1%	20 ± 2.52	15 ± 3.61

It observed from the results that the response of G88 which recorded higher results against the pathogenic bacteria due to the genetic properties of the verities. TiO_2_NPs may increase AgNPs' beneficial ability to enter the bacterial cell wall and connect to DNA and peptide groups, which in turn damages the cell wall, obstructs bacterial reproduction, and ultimately kills microorganisms^39,47,48^. Silver nanoparticles can stop infections by gathering in cell wall pores and causing cell membrane hydrolysis. These findings support those of Li et al.^49^ and El‐Naggar et al.^39^, they discovered that using of TiO_2_-AgNPs at a high concentration (0.6 g) is more advantageous for producing better antibacterial cotton.

### Cotton fabric's mechanical properties

#### Tensile elongation and strength

The Tensile elongation and strength data of the samples were collected in Table 5. Silver nanoparticles treatment with two different concentrations (0.01% and 0.1%) and succinic acid caused a change in fabric strength accompanied by a decrease in the concentration of Giza 88 which was recorded by 420.00 in the control sample decreased to 350.66 under concentration 0.01% then followed slightly increased by concentration 0.1% recorded of 360.00. In The case of Giza 94 also with decreasing in the concentration was recorded from 365.46 in the control sample to 291.06 in concentration of 0.01% followed by an increase under the concentration of 0.1% to 342.00, on the other hand, the case of core-shell TiO_2_-Ag in all varieties of the strength of the fabrics decrease again in the case of TiO_2_-Ag with concentration 0.01%. This results from succinic acid's acidity and the influence of TiO_2_, which reduced the tensile strength because TiO_2_ nanoparticles have greater oxidation and photocatalysis, and macromolecule linkages partially disintegrate under UV radiation. Through the development of a terminal group of fibrous formless molecular chains and the degradation of fibrous super-molecular structure, this ultimately reduced the fibrous macroscopic mechanical characteristics and caused damage to the fiber. According to Wang, et al.^50^, this increased in the case of 0.1 TiO_2_-Ag in all varieties as a result of the fabric's inter-yarn voids being filled, which caused friction between the yarns and made them resistant to stretching. However, with every increase in strength, fabric elongation decreases in the length of the specimen.

**Table 5:** Tensile strength and elongation of cotton fabrics G88 and G94.

Treatment	T.S		Elongation	
	G94	G88	G94	G88
Control	365.46 ± 0.87	420.00 ± 1.16	19.40 ± 1.33	18.13 ± 3.06
AgNPs 0.01%	291.06 ± 1.16	350.66 ± 1.16	27.29 ± 1.19	25.11 ± 1.16
AgNPs 0.1%	342.00 ± 1.16	360.00 ± 1.16	26.24 ± 1.18	23.07 ± 1.16
Ag-TiO_2_NPs 0.01%	330.26 ± 1.19	341.20 ± 1.17	22.32 ± 1.20	21.16 ± 1.17
Ag-TiO_2_NPs 0.1%	386.26 ± 1.19	473.00 ± 1.16	24.11 ± 1.16	22.02 ± 1.16

(T.S) Tensile strength as (N/f) elongation as % relative to control.

AgNPs and their core–shell nanoparticles treated with two different concentrations and succinic acid 5 g/L led to a decrease in the tensile strength which caused an increase in fabric elongation % in variety G94 which was increased by increasing the concentration relative to control that recorded 19.40, and increase in concentration (0.01% and 0.1% to (27.29 and 26.24) respectively, while the core–shell of nanoparticle (TiO_2_-Ag) with concentration (0.01% and 0.1%) the fabric elongation was decreased gradually recorded (22.32 and 24.11). The fabric elongation of G88 was increased in concentration (0.01% and 0.1%) and then decreased in the TiO_2_-Ag decreased the elongation of fabric owing to succinic acid's acidity and TiO_2_ which have photocatylic properties. These results in agreement with Akhavan et al.^51^ who found that the loss on tensile strength fabric could be related to the ultrasonic irradiation and/or cleavage of the cellulosic chains by acid hydrolysis.

TiO_2_ is one of the most effective photocatalytic materials, nano TiO_2_, is highly active, and has strong oxidizing strength and long-term stability. Electrons in nano TiO_2_ are excited from the valence band to the conduction band when exposed to ultraviolet light with wavelengths less than 385 nm. Hydroxyl radicals are formed when the positive hole in the valence band reacts with water or hydroxide ions adsorbed on the surface, Superoxide ions are formed when an electron in the conduction band reduces O_2_. These two highly reactive species can decompose a wide range of organic materials, including bacteria. With the aid of a binder, the TiO_2_ nanoparticles were effectively incorporated into cotton and maintained their antimicrobial activity for up to 10 washes^52–54^. TiO_2_ nanoparticles is the strong oxidizability of the photocatalyst, the fiber macromolecules can be easily broken so that the end group number of molecular chain of amorphous part of fiber increases and the super molecular structure of the fiber weakens. This finally results in a decrease in macro-mechanical property of the fibers. Thus, making the nanometer particle evenly disperse onto textile and tightly combining it with nanometer material and fiber shall be crucial during development and application for nanometer composite multifunctional textile^55,56^.

**Figure 11:**
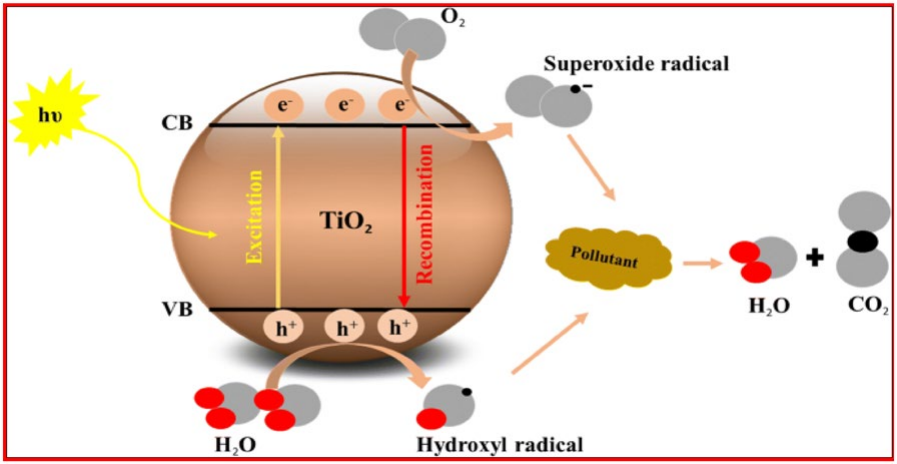
TiO_2_ semiconductor photocatalysis principle^63^.

Heterogeneous photocatalysis is predicated on the idea that when a semiconductor, usually TiO_2_, is absorbed, photon energy either equals or surpasses its band gap (hv ≥ Eg). Highly reactive oxygen species (ROS) are produced when the electron-hole pairs that are created participate in a sequence of oxidation-reduction events with species adsorbed on the TiO_2_ surface, as seen in Fig. 11. to produce oxygen species that are very reactive (ROS). These organisms interact with the microbes or adsorbed organic compounds on the TiO_2_ surface, causing them to break down into dangerous substances like CO_2_ and H_2_O. Nonetheless, electrons and holes have shorter half-lives, which frequently limits their availability for involvement in redox processes. Furthermore, energy released in the form of waste heat or light and a drop in process efficiency are signs of the recombination event. How can the lifespan of electrons and holes in TiO_2_ photocatalyst be extended before recombination occurs? is the challenge^57–62^.

**Figure 12:**
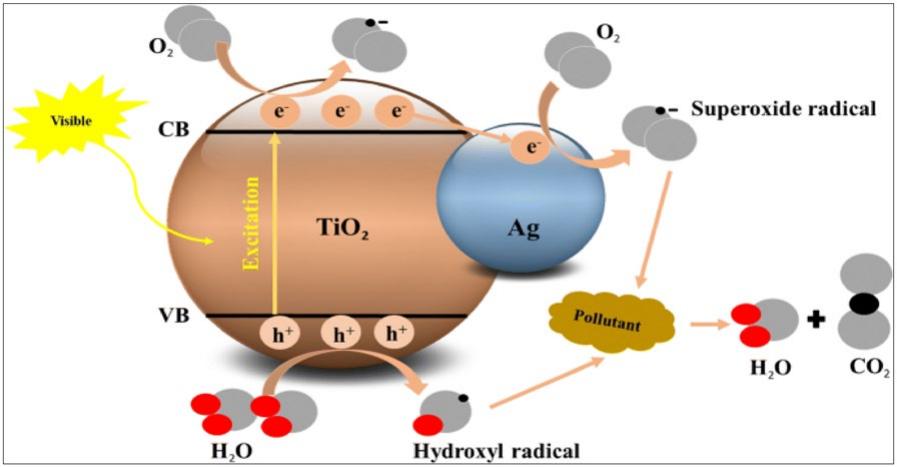
Photocatalytic activity of Ag/TiO_2_ photocatalyst^63^.

One of the effective methods for delaying the recombination of electron–hole pairs and shifting the light absorption range of TiO_2_ photocatalyst toward visible light is doping the material with silver nanoparticles^63,64^. Silver nanoparticles have two unique effects that increase TiO_2_ photocatalytic activity:

1. Serving as an electron trap to collect electrons moved from the TiO_2_ semiconductor's conduction band and move them to oxygen, whereupon they transform into superoxide radicals. Hydroxyl radicals are formed when water molecules combine with photogenerated holes in the valence band that is still present on TiO_2_. These free radicals are useful for inhibiting microorganisms and photocatalytically oxidizing contaminants (Fig. 12)^65^.
2. Producing the surface plasmon resonance (SPR) phenomenon, which simultaneously increases TiO_2_ photocatalytic effectiveness and expands light absorption into the visible light area^66,67^.

## Conclusions

In the current study, it was intended to synthesize functionalized AgNPs, and TiO_2_-AgNPs in straightforward, cost-effective, and reliable ways. AgNPs, and TiO_2_-AgNPs were prepared to be used in the treatment of cotton fabrics to improve their UV protection properties, self-cleaning, elongation and strength, as well as the antimicrobial activities to use the produced textiles for medical and laboratory uses and to increase protection for medical workers. The nanoparticles that had been created were confirmed to have a small size and good distributions. Additionally, SEM, zetasizer, and XRD analyses were used to study the chemical reactions between AgNPs and TiO_2_-AgNPs and the cotton fabric's succinate and cellulose chains. In comparison to the untreated cotton fabrics, treated cotton textiles showed improvements in surface roughness, elongation (%), and tensile strength. Functionalizing the fabric with TiO_2_-AgNPs led to an exceptional enhancement of treated cotton textiles. Additionally, cotton fabrics treated with TiO_2_-AgNPs have higher UPF ratings than fabrics treated with AgNPs and untreated cotton fabrics. Superior inhibitory activity was achieved by the antimicrobial cotton fabrics functionalized with TiO_2_-AgNPs against pathogenic bacteria including *S. aureus and E. coli*. The acquired results demonstrated that the AgNPs and TiO_2_-AgNPs covering offered UV protection and antibacterial action. This may be due to the nanoparticles' small size, which allows them to permeate materials with ease, as well as the beneficial effects of the crosslinking agent. As a result, cotton fabric treated with TiO_2_-AgNPs can be thought of as a versatile finishing agent effective for textiles used in sports and medicine.

## Data Availability

All data generated or analysed during this study are included in this published article.

## Abbreviations

AgNPs: Silver nanoparticles
DLS: Dynamic light scattering
G88: Giza 88
G94: Giza 94
OWF: On the weight of fabric
PS: Particle size
SEM–EDX: Scanning electron microscopy/energy-dispersive X-ray
SHP: Sodium hypophosphate
TEM: Transmission electron microscopy
TiO_2_-AgNPs: Titanium oxide-silver nanoparticles
TUV: Transmitted ultraviolet
UPF: Ultraviolet protection factor
UV–Vis: Ultra-violet visible spectroscopy
XRD: X-ray diffraction
ZP: Zeta potential
